# A Review of the Impact of Graphene Oxide on Cement Composites

**DOI:** 10.3390/nano15030216

**Published:** 2025-01-29

**Authors:** Ze-Yuan Hu, Yi Wan, Yan-Jun Duan, Ye-Hui Shi, Chun-Ping Gu, Rui Ma, Jian-Jun Dong, Dong Cui

**Affiliations:** 1 School of Safety Science and Engineering, Nanjing University of Science and Technology, Nanjing 210094, China; zeyuan_hu@163.com (Z.-Y.H.); dongjj@njust.edu.cn (J.-J.D.); 2 School of Physics, Nanjing University of Science and Technology, Nanjing 210094, China; wany@njust.edu.cn; 3 National-Local Joint Engineering Research Center for Biomass Materials of Electromechanical Product Packaging, Nanjing Forestry University, Nanjing 210037, China; duanyanjun@njfu.edu.cn; 4 Nanjing KENTOP Civil Engineering Co., Ltd., Nanjing 210041, China; shorce@163.com (Y.-H.S.); guchunping@zjut.edu.cn (C.-P.G.); 5 School of Civil Engineering, Zhejiang University of Technology, Hangzhou 310014, China; 6 School of Materials and Chemical Engineering, Anhui Jianzhu University, Hefei 230601, China; marui@ahjzu.edu.cn

**Keywords:** graphene oxide, cement composites, macro-microscopic performance, dispersion, reinforcing mechanisms

## Abstract

Graphene oxide (GO) has attracted significant attention as a nano-reinforcement for cement-based materials, owing to its exceptional mechanical properties and abundant surface functional groups. However, the precise mechanisms governing its effects in cement composites remain inadequately understood due to inconsistencies and gaps in the existing literature. This review conducts a comprehensive analysis of the dispersion and reinforcement effects of GO in cement materials, focusing on three key areas: (1) challenges associated with achieving uniform dispersion of GO in the high-pH environment of cement slurries and potential strategies to address them; (2) the influence of GO on the macroscopic properties of cementitious composites, including workability, load-bearing capacity, flexural strength, fracture resistance, and durability; and (3) the reinforcement mechanisms of GO, encompassing its role in hydration kinetics, alterations to the calcium-silicate-hydrate (C-S-H) structure, and bonding interactions at the cement matrix interface. Furthermore, recent advancements in optimizing the dispersion and reinforcement effects of GO, such as surface modification techniques, are explored, emphasizing its potential for multifunctional and intelligent applications. This review aims to provide engineering professionals with the latest insights into the application of graphene oxide as a nano-reinforcement in cement-based composites, while offering valuable guidance and direction for future research in this field.

## 1. Introduction

Cement is one of the most widely used construction materials globally due to its affordability and exceptional compressive strength. However, the inherent brittleness of concrete structures imposes limitations on its application in certain contexts. Calcium silicate hydrate (C-S-H) gel, the primary product of cement hydration, comprises nanocrystalline domains with atomic arrangements resembling tobermorite and/or jennite [1,2]. This unique characteristic offers valuable insights into leveraging nanomaterials and nanotechnology for enhancing cement composites, as nanoscale modifications to the microstructure can lead to significant improvements in macroscopic properties.

The advent of nanomaterials has presented transformative opportunities for enhancing the performance of cement-based composites. Research has demonstrated that the incorporation of nanoscale additives such as nano-ZnO, nano-silica, nano-Fe_3_O_4_, nano-TiO_2_, and carbon-based reinforcements like carbon fibers (CFs) and carbon nanotubes (CNTs) can significantly improve the mechanical properties and durability of cementitious materials [3,4,5,6,7,8]. The strengthening mechanisms of these nanomaterials are primarily attributed to two effects: the seeding effect and the filler effect. The extensive surface area of nanomaterials provides active centers for the growth of hydration products, ensuring their uniform distribution. Simultaneously, nanomaterials fill the pores within the cement matrix, creating a denser microstructure. Moreover, highly pozzolanic nano-silica reacts with calcium hydroxide (CH) to produce additional C-S-H gel, which is recognized as the most desirable hydration product of cement [4]. High-aspect-ratio nanomaterials such as CNTs and CFs contribute to improved structural integrity by arresting cracks, thereby inhibiting their propagation [7,9]. However, the practical application of these nanomaterials is often constrained by challenges such as strong van der Waals forces, which lead to high costs and difficulties in achieving uniform dispersion within the cement matrix.

Graphene, a single atomic layer of carbon arranged in a hexagonal lattice, represents a groundbreaking two-dimensional nanomaterial. In 2004, Andre Geim and Konstantin Novoselov isolated monolayer graphene from graphite through mechanical exfoliation, marking a significant milestone in nanomaterials research [10]. Graphene and its derivatives have garnered substantial attention due to their outstanding mechanical, thermal, and electrical properties [11,12,13,14]. Among these, graphene nanoplatelets (GNPs)—two-dimensional materials formed by stacking a few graphene layers—offer unique advantages. However, their chemical inertness and high hydrophobicity pose challenges for forming robust interfacial bonds with cement matrices [15]. Furthermore, intense van der Waals interactions between GNP particles often result in aggregation, limiting their uniform dispersion and diminishing their reinforcing effects. The use of reinforcements to enhance transverse connectivity in structural elements, such as prestressed concrete box girder bridges utilizing concrete-filled steel tube trusses (CFSTTs) and diaphragms, has shown significant improvements in stiffness and load distribution. These findings offer relevant insights for the design of nano-reinforced cement composites [16].

Graphene oxide (GO), a derivative of graphene, is typically synthesized by oxidizing natural graphite with strong oxidants and subsequently exfoliating it under ultrasonic conditions [17]. GO contains multiple oxygen-based functional groups, including hydroxyl, carboxyl, and epoxy groups, distributed across its surface and edges [18]. These functional groups reduce intermolecular forces and enhance electrostatic repulsion in aqueous solutions, improving GO’s dispersibility. Additionally, these groups provide active sites for further functionalization, thereby enhancing interfacial adhesion with the cement matrix [19]. Due to these advantages, GO has attracted considerable interest and has been widely investigated for its potential applications in cement-based composites [13,20,21,22].

In recent years, research efforts have increasingly focused on exploring the potential of graphene oxide as a nano-reinforcement material in cementitious composites. Despite notable progress, inconsistencies and contradictions in the literature have left the strengthening mechanisms and effects of GO incompletely understood. This study aims to summarize recent advancements in the reinforcement mechanisms and performance improvements of graphene oxide in cement composites, providing valuable guidance for engineers and a roadmap for future research.

## 2. Dispersion

To ensure the superior performance of graphene oxide as a nano-reinforcement material, it is crucial to achieve good distribution within the cement matrix. Although the electrostatic repulsion and hydrophilic properties of GO allow it to remain stable in aqueous solutions [23], achieving uniform dispersion in the alkaline pore solution of cement, which contains a high concentration of ions (Na^+^, K^+^, OH^−^, Ca^2+^, etc.), is a challenge [13,23,24,25]. Studies have shown that when GO suspensions are introduced into saturated Ca(OH)_2_ solutions, GO quickly aggregates as a result of the cross-linking interaction with Ca^2+^ [23,26]. Additionally, certain studies have pointed out that in highly alkaline environments, GO undergoes redox reactions, leading to a deoxygenation effect that further promotes its aggregation in the cement matrix [13,20]. The reduction in functional groups weakens repulsive forces among GO sheets and increases hydrophobic behavior, exacerbating the tendency for agglomeration.

The aggregation of GO in alkaline cement slurries not only hinders its performance as a nano-reinforcement material but also forms defect regions in the cement matrix, weakening the overall mechanical properties [27,28]. Therefore, exploring approaches to prevent GO aggregation is essential to fully realize its nanoscale additive functions.

**Figure 1:**
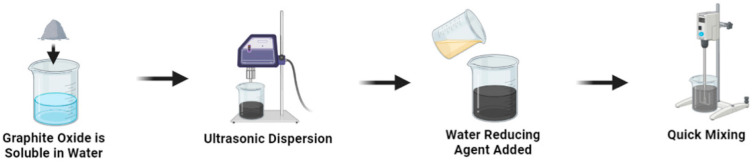
Preparation of graphene oxide suspension using ultrasound [31].

Dispersing nanomaterials in the form of suspensions can avoid the aggregation of powder in cement composites [28]. To prepare homogeneous GO aqueous suspensions, various physical and chemical approaches have been attempted, including ultrasound, electromagnetic stirring, intense shear mixing, the addition of dispersing agents, and surface modifications (covalent attachment or functionalization) [8,18,24,29,30]. Ultrasound is commonly employed to promote the dispersion of nanomaterials in aqueous solutions by applying mechanical force, as shown in Figure 1 [26,31,32].

Nonetheless, the limitations of these physical dispersion methods are that they only improve the distribution of GO in water. As a result of the strong interaction between carboxyl groups and calcium ions, significant aggregation still occurs in solutions containing Ca^2+^ [13,26]. Chemical modification involves attaching functional moieties onto GO surfaces or forming covalent linkages with agents, but this process is complex, time-consuming, and requires expensive equipment. It also introduces incompatible chemical substances into the hydration system, affecting hydration reactions [33,34].

Surfactants are a practical and simple approach for preparing well-dispersed nanomaterial suspensions, but certain surfactants used for dispersing carbon nanomaterials may exhibit incompatibility during cement hydration [35,36]. Recently, numerous studies have used compatible superplasticizers to enhance the distribution of nanomaterials in cement matrices [37,38,39,40]. These superplasticizers not only improve the dispersion of nanomaterials but also offset the reduction in fluidity caused by their incorporation.

The key to successfully dispersing nanomaterials is whether surfactants can adsorb onto their surfaces. Studies have shown that pH significantly affects the adsorption behavior. For example, Metaxa et al. [36] found that in alkaline conditions (pH > 10), the reduction of electrostatic repulsion diminishes the stability of surfactants on carbon nanomaterials, making it difficult for surfactants like SDBS and Triton X-100 to maintain their adsorption on carbon nanotubes, thereby reducing the strength characteristics of cement mortar. Miditana et al. [41] also observed that the photo-catalytic efficiency of TiO_2_ could be significantly improved by surface modification using Gemini surfactants in neutral and weakly alkaline conditions, but the adsorption effect of surfactants decreased as the pH increased. This is because the interaction between surfactants and nanomaterials weakens in alkaline environments, affecting their dispersion in aqueous solutions. Experimental results from Dalal et al. [42] showed that the adsorption of dispersants (such as SDS and SLES) on GO is reduced in alkaline environments, causing GO to re-aggregate in simulated cement pore solutions. Recent studies have demonstrated that poly-carboxylate superplasticizers (PCE) can prevent GO aggregation through electrostatic repulsion and steric hindrance [23,26,30]. This is because carboxylate molecules bond to the surface of GO, effectively inhibiting the formation of Ca^2+^ bridges between GO sheets [43]. However, Miao et al. [44] found that PCE was not effective in inhibiting GO aggregation in cement slurries. This discrepancy could be related to the unique molecular structure of PCE (such as charge density, side chain/main chain length, and polymer mass) and the properties of GO (such as flake dimensions, concentration, and oxygen content) [45].

The preparation techniques for cementitious materials with graphene oxide, as detailed in the existing literature, are presented in Table 1.

In most studies, GO aqueous dispersions are mixed with solid contents under the assistance of ultrasonic or mechanical stirring. However, even though GO sheets can disperse well in water, there is no guarantee of good distribution in cement pore solutions because of the interaction between calcium ions and the deprotonated carboxyl groups of GO [57]. Kaur and colleagues [13] found that the complex chemical environment of cement pore solutions (high ionic strength, high pH) exacerbates the stacking behavior of high-concentration GO sheets, forming large aggregates significantly exceeding the average 1 μm of GO sheets. Therefore, in these studies, the final performance of cementitious materials is influenced by the GO aggregates rather than the well-dispersed GO sheets.

In some studies, researchers have used silica fume (SF) to disperse GO in cement slurries [52,58]. Silica fume enhances the pozzolanic reaction, reducing calcium ion concentration and significantly enhancing the distribution of GO in cement slurries. However, there is no consensus on the distribution ability of GO in cement pore solutions. Xiong et al. [26] found that Ca^2+^ can form bridges between GO sheets, leading to aggregation.

Narimani et al. [59] studied the copolymer of acrylamide and 2-acrylamido-2-methylpropanesulfonic acid (AM-co-AMPS) modified GO. They found that the resulting (AM-co-AMPS)/GO significantly enhanced the flow characteristics of cement slurries while also exhibiting excellent dispersion stability and anti-loss properties in high-salinity and high-temperature environments. However, the chemical treatment of GO using copolymers is a complicated and lengthy procedure, rendering it impractical for industrial applications.

As shown in Table 1, the use of surfactants (especially PCE) in conjunction with ultrasound has been widely chosen to alleviate GO dispersion issues. The specific challenges and solutions for dispersing GO with PCE are outlined below:

(1) Optimizing the amount of PCE to avoid GO aggregation within the cementitious system. Research indicates that when the PCE:GO mass ratio is <1, the distribution of GO in cement pore solutions is significantly limited. However, increasing the PCE:GO mass ratio to 3–5 improves the stability of GO dispersion in the cement matrix [58]. Studies by Long [20] and Marbaniang [24] found that when the PCE/GO mass ratio is 10–20%, PCE coordinates with Ca^2+^ through its molecular chains, effectively inhibiting the bridging effect of Ca^2+^ on the GO surface and significantly enhancing dispersion. However, excessively high PCE concentrations can limit further interaction between GO and cement particles due to micelle formation, reducing dispersion efficiency. As a result, the amount of PCE should be considered an essential factor when preparing GO-containing cement composites.
(2) Optimizing the order of mixing cement, water, GO, and PCE. Research has shown that changing the mixing sequence can achieve two different states of GO dispersion: when GO and PCE are first mixed to form a suspension before being combined with cement, GO can disperse uniformly. Conversely, if GO is directly mixed with cement first (i.e., GO suspension is mixed with cement) and then PCE is added, GO aggregation occurs [60]. Similar phenomena have been observed by other researchers, where direct mixing of GO with cement results in a strong electrostatic shielding effect due to the chemical interaction between calcium ions and GO, limiting the adsorption and distribution effects of PCE molecular chains and causing irreversible GO aggregation [61]. Therefore, GO should be protected with PCE prior to its introduction into the cementitious mixture to prevent reactions with Ca^2+^.
(3) The dispersion efficiency of GO is closely associated with the molecular structure of PCE, particularly its side chains, backbone, charge density, polymer molecular weight, and functional moieties [23,26,62]. The dispersion of GO within cement pore solutions is also influenced by its flake size, oxygen content, and concentration [43,63]. As a result, carefully selecting the appropriate surfactant is crucial for achieving effective distribution of GO within the cementitious system.
(4) Verification experiments are suggested to confirm the dispersibility of GO within cement pore solutions. Although scanning electron microscopy (SEM) can provide detailed images of the microstructure of hardened cement composites after adding GO, studies have shown that SEM cannot fully capture the large-scale distribution of GO in the sample, especially in potential aggregation areas [64,65]. Moreover, due to the low dosage of GO (<0.1%), its small size, poor contrast with cement hydration products, and the limited resolution of SEM, it becomes challenging to distinguish GO nanosheets at the macroscopic scale in cement matrices [49,50]. Guo and colleagues [23] employed backscattered electron imaging (BSE) techniques to observe and assess the distribution of nanomaterials in cement pore solutions.
(5) The side effects of high dosages of superplasticizers should be noted. In low water-to-cement ratios or high nanomaterial content, additional superplasticizers are needed to achieve better dispersion. However, when PCE exceeds the saturation dosage, the slurry becomes overly fluid, increasing the risk of segregation and bleeding [66,67]. Studies have shown that excessive long side chains of PCE can cause increased slurry viscosity and reduced fluidity [68]. Therefore, developing advanced superplasticizers to more effectively distribute GO in cement pore solutions is imperative.

Through the above research, we can see that achieving consistent distribution of GO in the cement matrix requires a comprehensive consideration of multiple factors, including the dosage of PCE, the mixing sequence, the molecular structure, and the necessity of verification experiments. These methods provide important references and guidance for improving the application effects of GO in cement composites.

## 3. Performance

### 3.1. Workability

Workability refers to the ease with which new cement composites flow and compact, playing a crucial role in the structural performance and long-term resilience of hardened cementitious materials. Insufficient flowability can render it difficult to compact fresh cement composites, exacerbating the retention of air voids, and ultimately weakening the material’s mechanical properties [28].

Many studies have shown that incorporating graphene oxide negatively impacts workability, as indicated in Table 2. The hydrophilic functional groups on the GO surface, along with its extensive surface area easily lead to the adsorption of a significant amount of water molecules in aqueous systems, thereby reducing the lubrication effect of cement particles and increasing the frictional resistance between them [37,38,56,69,70]. The high dispersibility and hydrophilicity of GO significantly impair the flowability of cement composites because its extensive surface area requires a substantial amount of free water to adhere to and maintain the dispersed state [30,71]. Some studies have found that the formation of GO aggregates in the slurry increases the free water capture area, further reducing the fluidity of cementitious materials. The physical interactions between GO and cement particles further explain the limitations on slurry flowability, as the van der Waals forces among GO layers enhance the attraction between cement particles [12,32].

**Figure 2:**
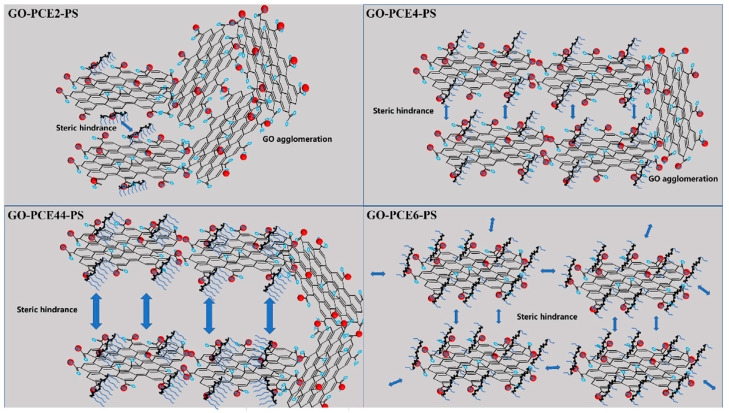
Mechanism of improved dispersion by GO-PCEn in the cement pore solution [45].

**Figure 3:**
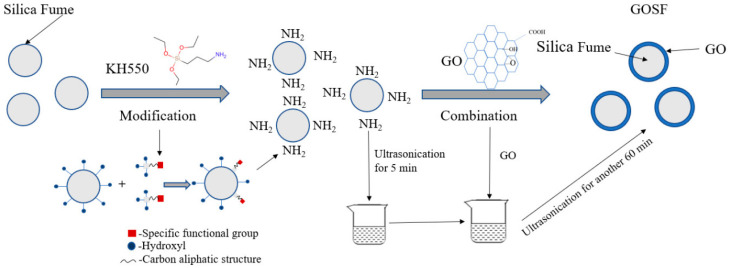
The preparation principle of GOSF [58].

Several methods have been proposed to address the adverse effects of GO on workability. Adding poly-carboxylate superplasticizers (PCE) is considered the most practical and efficient approach to solving this issue [13,22,23,62,77,78,79]. PCE optimizes particle dispersion through electrostatic repulsion and steric hindrance and significantly reduces the retention of free water, thus compensating for the decrease in water content due to the inclusion of GO [30]. Qin et al. [45] found through their research that PCE with longer side chains improves the dispersion of GO in cement, as shown in Figure 2. For a constant side chain length, raising the charge density enhances dispersion; similarly, for a constant charge density, extending the side chain length can effectively improve dispersion efficiency. It has been reported, as shown in Figure 3, that adding graphene oxide-coated silica fume (GOSF) reduces the flocculation effect between particles and decreases the plastic viscosity and rheological parameters of the slurry [39,75]. Similarly, fly ash (FA) can reduce internal friction in the slurry through the ball effect, thereby improving the flowability of fresh cement slurries [19]. In some studies, nanocomposites have been synthesized by chemically reacting GO with carboxymethyl chitosan [80], polyacrylamide (AM) and sulfonated monomers [59], methyl palmitate (MPA) [81], PCE monomers [26], and other chemical agents to improve the rheological behavior and flowability of cement slurries.

### 3.2. Mechanical Properties

In most applications, the load-bearing capacity of cementitious materials is regarded as the most crucial property. Lately, numerous studies have explored the capability of graphene oxide as a promising reinforcing material to enhance the structural performance of cementitious composites, as shown in Table 3.

Research has shown that adding small doses of GO to cement composites can significantly enhance their strength in compression, bending, and tension. The proposed mechanisms in the literature are as follows: (1) Graphene oxide itself possesses excellent mechanical properties, leading to a significant reinforcing effect on cement composites [63,75,84,85]; (2) GO can promote the development of ettringite and calcium silicate hydrate, thereby enhancing the structural stability of the composites [73]; (3) the templating effect of GO creates active centers for the growth of hydration phases, resulting in a denser microstructure and inhibiting crack propagation in the initial stage [22,31,86]; and (4) improved interfacial properties: GO chemically bonds with the cement matrix, improving the interfacial performance and enhancing the efficiency of load transfer [19,87]. The alteration of the microstructure of cement composites by graphene oxide is the primary factor influencing the overall properties of cement composites, and this will be discussed in detail in Section 4.

In these reports, there are significant differences in the growth rate and optimal dosage of GO. The factors affecting the reinforcement effects of GO include its characteristics (such as flake size, oxygen concentration, and number of layers) and mechanical properties, as well as the type of cement, high-efficiency water reducers, water-cement ratio, curing conditions, curing age, and the method used to prepare GO-cement composites.

In certain studies, GO suspension was added directly to cement without prior modification, and the improvement in mechanical strength may have been due to the aggregation of GO rather than the dispersion of GO nanosheets. Fonseka et al. [30] found that 0.08 wt% GO aggregates can provide a high-strength reinforcement effect, increasing compressive, flexural, and tensile strengths by 21%, 23%, and 12%, respectively. Malak et al. [88] reported that pre-stabilized GO, which enhances interfacial adhesion and reduces aggregation, significantly improves the strength and performance of cement paste compared to GO added later. Hong et al. [49] also found similar results, indicating that the compressive and bending strengths of cement composites are greater when PCE-modified GO is mixed with cement compared to unmodified GO directly mixed with cement. Edwards et al. [12] found that well-dispersed GO can significantly enhance the binding strength of calcium silicate hydrate within the cement matrix, thereby enhancing the structural performance of cementitious materials. Yu and colleagues [46] examined the influence of GO aggregate size on the reinforcement ability of cement paste and discovered that the more pronounced the GO aggregation, the lower the compressive and bending strengths of cementitious materials. This is because of the reduced specific surface area, which prevents the formation of a reinforcing interface with the cement matrix. Therefore, despite some studies showing that bonded graphene oxide improves the load-bearing capacity of cementitious composites, it is plausible to suggest that larger GO nanosheets (following proper modification) can promote multiple nucleation and cross-linking of C-S-H, thereby enhancing the strength characteristics of cementitious materials [18].

Numerous studies have analyzed how GO concentration, oxygen levels, sheet dimensions, and other factors on its reinforcement effect in cement composites. Ren and colleagues [76] investigated the reinforcing effects of five different GO concentrations (0.00%, 0.02%, 0.04%, 0.06%, and 0.08% by cement weight) on cement-based composites. Jiang et al. [75] found that the most significant increases in compressive and flexural strengths occur when the GO content is 0.06 wt%, and further increases in GO content result in a decrease in strength because of aggregation. Other studies have also found similar trends [31,38,85]. For example, Wang et al. [89] found that at higher concentrations, the performance of cementitious materials decreases due to the overlap of GO sheets and the propagation of micro-cracks. When the GO concentration is 0.09 wt%, the compressive strength of geopolymer foam concrete increases by 58.37%, but after further concentration increases, the 30-day strength decreases due to the difficulty in uniformly dispersing GO.

Kaur et al. [13] found that decreasing the GO size via ball milling improves its reinforcement capabilities within the cement matrix, consequently increasing the load-bearing capacity of cementitious materials. Smaller GO sizes increase the specific surface area, providing additional centers for the growth of hydration phases, which further promotes the hydration process [50,90]. Fonseka et al. [18] also reported the same trend; researchers used smaller GO particle sizes (43–60 µm raw material) and observed an 11.8% increase in early (7-day) compressive strength, while larger GO did not show the same improvement. However, Mao et al. [91] found the opposite trend, discovering that larger GO sheets, due to their higher defect density and oxygen content, can provide additional sites for hydration product formation, resulting in a more compact microstructure and significantly improved compressive and tensile properties.

Researchers have also attempted other methods to enhance the reinforcement effects of GO in cement composites. According to reports, Li et al. [78] used a silane coupling agent (MPS) to modify the surface of GO and coat it on the surface of recycled rubber aggregate (RRA). The modified RRA mortar showed significantly improved interfacial performance. Compared to unmodified GO, the compressive strength of the modified composite was enhanced by 19%, and water absorption and chloride ion diffusion resistance were also significantly improved. Qiao et al. [92] enhanced the bonding between carbon fibers and the cement matrix by depositing GO on carbon fiber surfaces through electrophoretic deposition. This method provided more nucleation sites, and compared to ordinary samples, it significantly increased the compressive strength (5.04%) and flexural strength (25.53%).

The incorporation of GO not only enhances the compressive, flexural, and tensile capacities of cementitious materials but also significantly improves other mechanical properties, including dynamic elastic modulus, deformation capability, and toughness. Additionally, the addition of GO optimizes dynamic mechanical parameters, including reducing the loss factor, increasing the storage modulus, and energy absorption performance, thereby further improving the overall mechanical performance and durability of the materials [22,54,72,75,83,89]. Many researchers have introduced GO into geopolymers [93,94,95,96] and other special binders, such as ultra-fine slag [97] and fly ash-based [98] systems. The findings indicate that the mechanical properties of the materials are enhanced to varying degrees after introducing GO. Furthermore, studies have proven that incorporating GO could facilitate the efficient use of recycled aggregates and waste cement in engineering, effectively reducing environmental burdens and supporting the sustainable development of the construction industry [31,53,78,99].

The recent literature indicates that GO can enhance the structural performance of cementitious materials. Nevertheless, existing research primarily examines the behavior of GO in cement pastes or mortars with elevated water-cement ratios, and studies on the strengthening impact of GO in ultra-high-performance concrete with low water-to-binder proportions are scarce. Furthermore, research on the structural applications of GO is scarce. Therefore, more detailed experimental studies are needed to investigate the reinforcement effectiveness of GO in concrete with very low water-to-binder ratios or at larger scales to facilitate its practical application in construction.

### 3.3. Durability

The long-term performance of cementitious materials pertains to their capacity to maintain structural integrity and resist the penetration of corrosive ions (such as CO_2_, SO_4_^2−^, and Cl^−^) under conditions of chemical corrosion and periodic wet-dry cycles [100]. By enhancing the durability of cement composites, not only can their service life be effectively extended, but maintenance and repair costs can also be significantly reduced. Compared to comprehensive studies on mechanical properties, research regarding the durability of GO-enhanced cementitious materials is less extensive. This section summarizes the impact of graphene oxide on the long-term resilience of cementitious composites as reported in current studies.

The transport properties refer to the speed at which corrosive substances (such as water, sulfates, and chlorides) penetrate into the internal structure of cement composites from the external environment. Cracks and pores are the primary pathways for the propagation and diffusion of corrosive agents in cement composites [89]. Djenaoucine et al. [101] found that 0.005% graphene oxide can refine the microstructure of concrete, effectively inhibiting the penetration of chloride ions and thus protecting the embedded steel from degradation. Another study reported that when the pH decreased from 12 to 9, the chloride binding capacity of cement pastes with 0.1 wt% GO increased by approximately 31%, indicating a notable enhancement in the durability of cement composites [87]. These results align with those of Liao and colleagues [19], who demonstrated that the synergistic addition of FA and graphene oxide performs better in resisting chloride ion penetration, with the resistance to soaking coefficient of ordinary cement paste increasing from 0.78 to 1.007. Additionally, Lu et al. [72] developed a new composite material of graphene oxide and recycled fine aggregate (WGO@RFA), which significantly improved the durability of concrete by increasing the resistance to water molecule migration and ion transport within the mortar.

Furthermore, GO applied as a surface coating on concrete can significantly enhance its resistance to chloride ion diffusion and carbonation, effectively extending the service life of concrete [102]. Some researchers have also investigated the freeze-thaw durability of cement-based materials with graphene oxide. Wang et al. [89] found that small doses of GO (such as 0.03 wt%) can refine the pore network and reduce the number of interconnected voids, thereby increasing the matrix’s density and inhibiting water transport, which improves the freeze-thaw resistance of concrete. Cui et al. [103] further supported this by showing that GO incorporation reduces the average porosity of mortars by 3.37–11%, which contributes to the improved leaching resistance. Another study, by analyzing the loss of dynamic elastic modulus and compressive strength, found that specimens with 0.03 wt% GO showed better freeze-thaw resistance, with a more spatially uniform pore distribution [104]. These results align with the observations made by Zhang and colleagues [22], who demonstrated that incorporating GO notably increased the density of concrete, with the initial and final freeze–thaw strength of concrete containing 0.05% GO increasing by 23.3% and 41.9%, respectively, and enhanced the material’s durability by promoting early hydration reactions.

When cement composites come into contact with corrosive solutions with pH values below 12.5, the calcium is typically leached out. The calcium leaching leads to the breakdown of CH and C-S-H gels, which increases pore connectivity and weakens the strength of concrete. Research has shown that incorporating graphene oxide could significantly mitigate the leaching of calcium in cement composites under low pH conditions by refining the pore structure [13]. Cui et al. [103] confirmed this by using a novel dual-scan method to demonstrate that GO incorporation enhances both the leaching and cracking resistance of mortar, which is crucial for maintaining the durability of cementitious composites in aggressive environments. This conclusion is supported by multiple studies. For example, Wang and colleagues [105] discovered that the inclusion of GO films significantly decreased porosity and pore throat diameter, optimized the microstructure of calcium silicate hydrate, and enhanced the ability of cementitious composites to resist calcium leaching in aggressive aqueous media.

These studies indicate that GO-reinforced cement composites exhibit good durability, but to more effectively incorporate GO into practical construction practices, further research is needed on the long-term performance of cement-based materials under sulfate attack, steel corrosion, alkali-aggregate reactions, and other conditions.

## 4. Mechanism

To investigate the mechanisms by which graphene oxide improves the structural performance and long-term resilience of cementitious materials, numerous studies have conducted microscopic studies using various characterization methods and instruments. The subsequent sections provide an overview of recent studies on the impact of graphene oxide on the microstructure of cementitious materials, highlighting areas such as hydration dynamics, calcium silicate hydrate formation, porosity, and interfacial interactions.

### 4.1. Hydration Kinetics

Cement is a powdery substance that reacts with water to form various intricate hydration products, including calcium silicate hydrate gel, CH (calcium hydroxide), AFt, and AFm. The formation of these hydration products is significantly influenced by hydration kinetics, which is critical in shaping the evolution of the microstructure and ultimate performance of hardened cementitious materials [106]. It is generally believed that active graphene oxide nanosheets act as active centers for the growth of hydration phases, thus accelerating the hydration of cement. Nevertheless, the precise role of GO in the hydration mechanism remains unclear because of the conflicting experimental findings found in existing studies.

**Figure 4:**
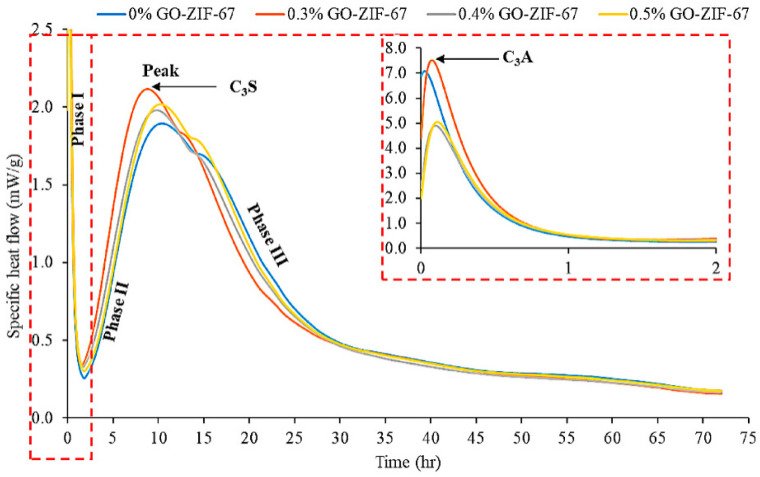
Specific heat flow of nanocomposite cement mortar during hydration [107].

**Figure 5:**
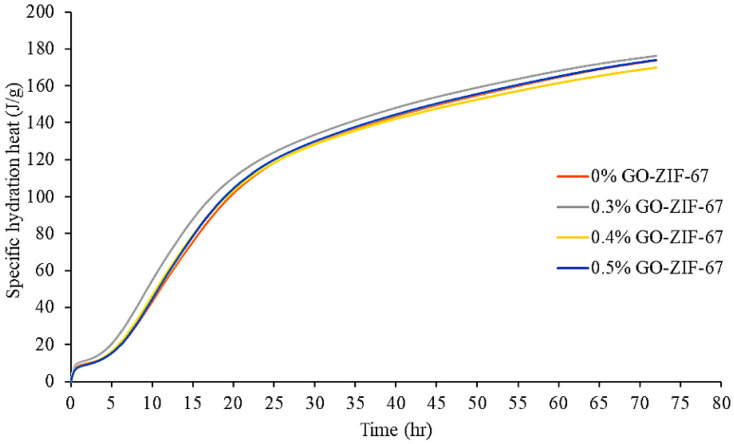
Specific heat generation of nanocomposite cement mortar [107].

Many researchers have employed isothermal calorimetry to investigate the influence of GO on cement hydration. The results show that cement samples containing GO exhibit notably enhanced cumulative hydration heat compared to ordinary cement, indicating that GO accelerates hydration and produces more hydration products [106]. Al-Fakih et al. [107] conducted thermal gravimetric analysis (TGA) on nano-composite cement mortars containing graphene oxide encapsulated in zeolitic imidazolate framework-67 (GO@ZIF-67) and found that the endothermic process caused by the decomposition of the reactive components due to temperature rise (Figure 4) is related to the weight loss observed in the samples (Figure 5). The exothermic peak and total heat release of the GO@ZIF-67 composite materials were significantly increased, suggesting that well-dispersed GO@ZIF-67 can provide more nucleation sites, thereby promoting the hydration reaction. Similar phenomena have been observed by other researchers [108]. However, Qin et al. [109] found that the addition of polycarboxylate-based superplasticizers (PCE) did not significantly change the evolution release during cement hydration. This inconsistency suggests that the type and dosage of dispersants, as well as the characteristics of GO, are critical factors in the hydration of cement.

Compared to the reference sample (IGOC-0), Gan and colleagues [106] observed that the total hydration heat released by cement paste with IGOC-33 increased by 7.69% after 72 h, and the hydration heat peak occurred earlier, indicating that the large specific surface area of IGOC-33 accelerates the hydration process. Ajith et al. [110] found through various characterization techniques that GO binds to carbon-based surfaces through π-π stacking and hydrogen bonding, forming a multi-layer adsorption structure and exhibiting excellent adsorption capacity. However, Ayora-Gutiérrez et al. [111] found through X-ray spectroscopic analysis that unmodified GO cannot effectively adsorb on certain inorganic matrices.

The content of bound water (NEW) and CH in cement paste, typically derived from TGA curves, is often employed as a key metric to measure the degree of cement hydration. The three main stages of mass loss in TGA curves are: from ambient temperature to 150 °C, associated with the evaporation of water in calcium silicate hydrate gel and free water in pores; from 400–500 °C, corresponding to the decomposition of CH; and from 600–800 °C, due to the decarbonation of CaCO_3_ caused by uncontrolled carbonation [40,54,107,112,113]. The content of non-evaporable water is usually determined by the mass loss percentage between 105–1000 °C [97] or 145–1000 °C [90].

Zhao et al. [40] observed a 13.36% increase in the content of NEW and the content of CH decreased by 12.64% in cement mortar containing GO compared to the reference sample, suggesting that the addition of GO promotes the hydration degree of cement, leading to more significant hydration. Studies have shown that the content of calcium silicate hydrate increased to 20.759% in cement samples containing 0.04 wt% GO, suggesting that GO promotes the cement’s hydration level by increasing the number of nucleation sites [39]. This result aligns with the findings of Nithurshan and colleagues [25]. Miao and colleagues [44] found that incorporating the GO-NS mixture increased the orientation index of CH and the polymerization level of calcium silicate hydrate gel during the early hydration stage, proving that GO-NS exhibits significant hydration promotion as a nano-additive in cement. However, in Yu and colleagues’ research [39], the calcium silicate hydrate content in cement paste containing 0.06 wt% GO not only failed to increase but actually decreased, possibly due to the aggregation of excess GO, which affected the nucleation effect.

Guo and colleagues [23] discovered that adding graphene oxide lowered the CH content while boosting the content of C-S-H. They explained that the templating effect of GO modulates the crystal structure of hydration phases, enhancing the polymerization level of calcium silicate hydrate during the initial hydration phase [114]. Cheng and colleagues [31] discovered that the trend of CH content changes in cement paste is complex with the addition of GO. The amount of CH steadily rose as the GO content ranged from 0 to 0.06 wt%, but decreased when the GO content surpassed 0.06 wt%. At a GO content of 0.08 wt%, the CH content was merely 7.14% greater than that of cement paste without GO. This paper suggests that high content and large specific surface area of graphene oxide may hinder cement hydration by excessive amounts of water. On the other hand, some researchers believe that GO acts as a nucleation site for adsorbed water molecules, promoting the migration and reaction of water molecules, thus aiding the hydration mechanism [109].

X-ray diffraction (XRD) analysis is utilized to study crystalline structures, enabling the evaluation of hydration reactions and hydration degree. Studies show that after the addition of GO to cement composites, the overall crystallinity of the XRD patterns did not significantly change compared to the reference samples, indicating that GO has limited influence on the main crystalline phases of the cement matrix [14,31,39,115]. However, some research has shown that the addition of graphene oxide results in a decrease in the peak intensity of alite (C_3_S) and belite (C_2_S) in cement components, while the peak intensity of calcium hydroxide rose significantly, suggesting that the presence of GO promotes the cement hydration process [23,76,116,117]. Yang et al. [57] observed that during the early stage of cement hydration, the carboxyl groups (-COOH) on the GO surface form complexes with Ca^2+^ on cement particles, which induces and accelerates the formation of hydration phases, aiding the growth of hydration crystal products. These findings suggest that GO not only enhances the hydration process of cement but significantly contributes to the formation of hydration products and the optimization of micro-structure through its functional groups.

**Figure 6:**
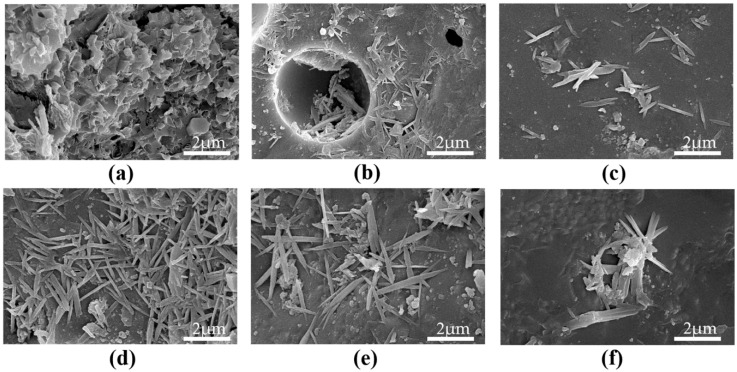
SEM images of different kinds of mix proportions at 28 days: (**a**) GO-0; (**b**) GO-2; (**c**) GO-4; (**d**) GO-5; (**e**) GO-6; (**f**) GO-8 [120].

Many studies have used SEM to observe how graphene oxide influences the microstructure characteristics of cement hydration phases. SEM micrographs show that under the presence of GO, hydration phases are more evenly distributed and the structure is more dense, which may be ascribed to GO’s seeding effect, promoting the growth of hydration products [54,105,114]. Additionally, some fine cracks, mostly branched rather than through cracks, can be observed in SEM images, indicating that GO helps delay crack propagation by regulating the structure and form of hydration products [19,73,118,119]. Wang et al. proposed through SEM studies that GO nanosheets could serve as templates for the formation of hydration phases, regulating the creation of flower-like structures [83,86,105], as illustrated in Figure 6, leading to more regular and dense morphologies. However, Gong et al. further found through SEM-EDS analysis that the primary chemical component of some flower-like crystals formed in cement is calcium carbonate (CaCO_3_). This phenomenon suggests that incorporating graphene oxide may induce the nucleation and ordered growth of calcium carbonate crystals, rather than simply affecting hydration products. Due to the potential interference from carbonation, the specific mechanism of GO in influencing cement hydration products still requires more in-depth research to clarify.

### 4.2. C-S-H Structure

Studies on the alterations induced by graphene oxide in the calcium silicate hydrate structure at the nanoscale have attracted significant interest because C-S-H consists of nanocrystal-line regions with an atomic structure similar to that of tobermorite and/or jennite [2,121].

Using nanoindentation techniques to measure the stiffness of each test group, the results show that the proportion of low-density C-S-H in GO-cement composites significantly decreased, while the quantity of high-density calcium silicate hydrate increased relative to the reference sample. This finding indicates that GO leads to the formation of a greater amount of high-density calcium silicate hydrate, resulting in a denser cementitious matrix [40].

Nuclear magnetic resonance (NMR) spectroscopy is often employed to examine the chemical structure and polymerization characteristics of calcium silicate hydrate, where Q^n^ represents the types of silicate structures (*n* = 0–4), with Q represents the silicate configurations (*n* = 0–4), with Q representing silicate tetrahedra and n indicating the number of oxygen atoms interacting with adjacent tetrahedra. The average chain length (MCL) is used as a metric to quantify the polymerization level of calcium silicate hydrate, reflecting the extensibility of silicate chains [25]. According to the study by Suh et al. [85], Q^0^ phases of C_3_S and C_2_S, along with Q^1^ and Q^2^ phases of C-S-H gel, appeared in the spectra, indicating that GO speeds up the hydration process of silicate phases. GO added to a calcium-rich environment induces the development of calcium-rich calcium silicate hydrate phases on its surface, while silicon-rich phases form near the GO sheets.

GO promotes the polymerization of C-S-H structures through its reactive groups on the surface, which interact with Ca^2+^. Gong et al. [113] found in NMR spectra that Q^1^ and Q^2^ were 29.1% and 39.4%, respectively, in cement paste doped with GO, compared to 27.6% and 28.1% in the control group, suggesting that GO enhances the formation of calcium silicate hydrate and increases the polymerization degree of silicates.

Another study showed that the trend of total signal intensity changes was very similar across all pastes, and the signal intensity decreased with increasing GO content, with the critical point also appearing earlier. This phenomenon further confirms the promoting effect of GO in cement hydration [31]. The study by Meng and colleagues [112] demonstrated that the ability of GO sheets to serve as nucleation sites led to a 51.6% increase in MCL, significantly enhancing the polymerization extent of calcium silicate hydrate. Additionally, studies have reported that while the addition of GO improves the hydration degree, its other effects on the C-S-H structure are limited [109]. Therefore, further research on the mechanism by which GO regulates the nanostructure of C-S-H remains essential.

### 4.3. Pore Structure

Cementitious materials contain numerous capillary voids and pores, and the pore configuration is considered a critical parameter closely related to mechanical and durability properties. Under low workability conditions, poor compaction of new cement-based composites is associated with trapped voids. Capillary pores can be seen as the remaining spaces initially filled by water, which could be further categorized into gel pores (2.5–10 nm), intermediate pores (10–100 nm), capillary pores (100–1000 nm), and large pores (exceeding 1000 nm) [14,26].

The porosity characteristics of cementitious composites enhanced with graphene oxide have been extensively studied through mercury intrusion porosimetry (MIP) and gas adsorption methods. The findings indicate that the presence of GO significantly reduces the overall porosity of cementitious materials, resulting in a denser microstructure and thereby improving their mechanical properties [18,31,55,72,73,84,85]. Researchers have suggested two mechanisms for the refinement of the pore network through GO: one mechanism is the pore-filling action of nano GO sheets, which offer more nucleation sites during the hydration process, leading to a finer pore structure [21,39,40,95,122]; the other is the seed effect of GO, which encourages the formation of hydration phases, thereby filling the pore spaces [1,12,14,26].

Notably, the pore size distribution provides more detailed insights into the porosity characteristics of cementitious materials than the total porosity. By examining the differential mercury intrusion curves along with the total pore volume curves of the samples, the pore structure of mortars containing GO sheets can be better revealed. The research found that incorporating GO decreased the average pore diameter of the mortar, and compared to the control samples, its cumulative pore volume decreased by more than 12%, while the capillary pore fraction increased. This can be attributed to the addition of well-dispersed nano GO particles [12]. Yu et al. [39] conducted MIP tests and found that as the GO content rose to 0.04%, the volume of pores in the size range of 100–1000 nm significantly decreased from 1.8% to 1.1%, indicating that GO can refine the pore structure by enhancing the hydration reaction and its nanofiller effect. Suh et al. [21] discovered that GO addition decreased the formation of pores exceeding 50 nm and prevented the interconnection of pores, making the micro-structure of the matrix more dense. Kaur et al. [13] studied the pore structure changes induced by GO by adding different amounts of GO to FA-cement mortar. The cumulative pore volume graph showed that the addition of GO did not reduce but rather increased the specific surface area of pores in cement composites, which is a sign of pore refinement. This is because, as shown in the literature [12], higher doses of GO may lead to additional GO nanosheets agglomerating and sliding under stress, partially offsetting the benefits of reduced pore volume.

Safarkhani et al. [74] conducted MIP tests and found that well-dispersed GO not only significantly reduced the critical pore diameter and total pore volume in cement but also enhanced the fraction of gel pores. The formation of gel pores is attributed to the generation of additional hydration phases like calcium silicate hydrate. Many other researchers have also confirmed this increase in gel pores. For example, silane-functionalized graphene oxide nanocomposites (GO-APTS) effectively filled internal pores in cement paste, shifting the maximum of the pore size distribution curve from the 0.1–1 μm range to the 0.01–0.1 μm range of smaller pores [95]; Nithurshan et al. [25] found that incorporating GO transformed the gel pore diameter in hydration products from 5.52 nm to 3.40 nm, which is likely due to the creation of more ordered calcium silicate hydrate by GO, transitioning C-S-H from a “lower density” to a “higher density” structure. At the same time, it was found that incorporating GO increased the hydration extent, with the formation of hydration phases filling the pore network and decreasing the total pore volume. A hypothesis was proposed that nano GO could occupy the gel pores in C-S-H gel via an “intercalation mechanism” [123].

These contradictory conclusions in the studies could be affected by several factors, such as the analytical tools and approaches, the water-to-binder ratio, and the properties of GO, particularly its distribution within the cement matrix. The refinement of the pore network in GO-modified cementitious materials is the most critical factor affecting their mechanical and durability performance, and therefore, further research is needed to clarify its specific mechanisms and optimization conditions.

### 4.4. Interface Bonding

To successfully improve the properties of cement-based materials using nanomaterials, two important conditions need to be fulfilled: proper dispersion and sufficient interfacial bonding [26]. When robust interactions are formed, the stress can be efficiently transferred through the interface to the reinforced nanomaterial regions, thereby reducing localized stress concentrations and improving overall mechanical properties. However, if effective chemical bonding does not take place between the nanomaterials and the matrix, a weak interface may result, leading to the nanomaterials slipping out of the matrix under load and failing to fully exert their mechanical reinforcement effects, or even negatively impacting the strength and toughness of the composite material [124].

**Figure 7:**
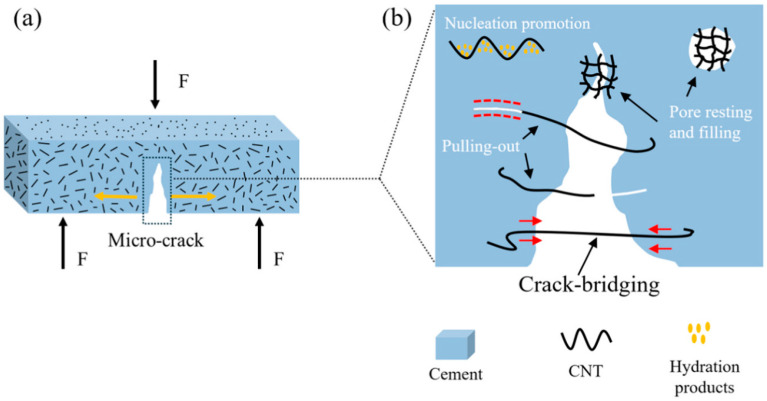
Diagram of the reinforcing mechanisms of MWCNTs-OH in cement-based composites (**a**) Three mechanisms of interaction between individual MWCNT-OH and cement matrix under mechanical load. (**b**) Pull-out and detachment behaviors depending on the relative strength of MWCNT-OH and bonding strength with the cement matrix [7].

The water-repellent characteristics of carbon nanotubes hinder their ability to form good interfacial bonding with cementitious composites. To overcome this limitation, Yan et al. [7] modified CNTs by attaching carboxyl functional groups to their surfaces. FT-IR analysis confirmed the effectiveness of the modification of multi-walled carbon nanotubes (MWCNTs) and the enhanced mechanism (as shown in Figure 7), revealing three different failure modes and their comprehensive contribution to mechanical performance, which elucidated the critical role of MWCNT-OH in improving interfacial load transfer efficiency and inhibiting crack propagation. Researchers found that when PCE polymers were added to cement paste, the large number of carboxyl groups in the polymer strongly bound metal ions like Ca^2+^ in the solution, forming R-COO-Ca^2+^ complexes to stabilize the early-formed AFt and C-S-H phases [62]. Molecular dynamics simulations further revealed the mechanisms of interplay between the functional moieties of graphene oxide and calcium silicate hydrate [125]. The study showed that the -COOH functional groups in GO form strong electrostatic interactions with Ca^2+^ ions, stabilizing C-S-H atoms onto the GO surface, thereby significantly enhancing the integrity of the calcium silicate hydrate structure.

It is suggested that analogous interactions may occur between the functional moieties of GO and the cementitious matrix, conferring enhanced mechanical strength and durability to the cementitious material [12,22,24,26,43,100,104]. In certain investigations, the interactions between GO and the cement matrix were characterized using SEM images [12,14,53,71]. However, the evidence from SEM images regarding the adhesion of GO to the cement matrix is not convincing, as the limited amount of GO and the low contrast between GO and cement hydration products make it challenging to detect nanoscale GO flakes in cement-based materials. Additionally, there are doubts about whether SEM images can adequately fulfill the requirements for assessing the adhesion at the graphene oxide/matrix interface. To further investigate the interactions between GO and the cement matrix, it is essential to incorporate additional characterization methods.

**Figure 8:**
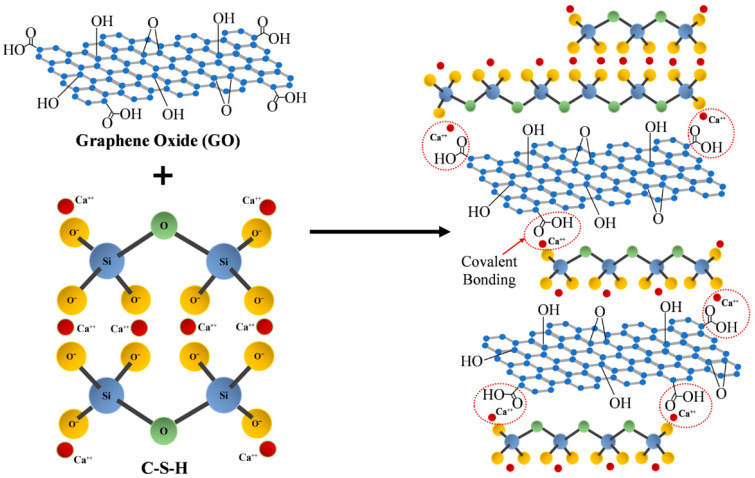
The formation of the three-dimensional grid structure in GO-added concrete [18].

Fonseka and colleagues [123] investigated the interactions between GO and cement composites. In cement paste containing GO, the carboxyl moieties on the GO surface can interact with Ca^2+^ ions in the pore solution, resulting in the formation of a three-dimensional network structure linked by -COO-Ca-OOC-. This allows the hydration products to develop on the GO surface. The three-dimensional network improves the connectivity between hydration phases, known as the “bridging mechanism” of GO, stabilizing the calcium silicate hydrate molecules (as depicted in Figure 8). Some investigators used reactive molecular dynamics simulations to gain a deeper insight into the interactions between GO and the cement matrix. The findings showed that the deprotonated carboxyl moieties of GO chemically react with calcium silicate hydrate gel via Ca^2+^ coordination and hydrogen bonding [126].

The stability of GO functional groups throughout the cement hydration process is a key factor in ensuring strong interfacial bonding with the cement matrix. Long and colleagues [87] discovered that incorporating GO significantly increased the capacity of the cement material to bind chloride ions, especially in low-alkaline environments where the pH value decreases to 9. This characteristic is attributed to the chemical cross-linking effect of Friedel’s salt (FS) promoted by GO, which interacts with the surface groups of GO and Ca^2+^ ions. Furthermore, SEM analysis showed that a higher GO content encouraged the development of flower-like overlapping sheet structures of FS crystals, further enhancing the chemical binding capacity of chloride ions. The dispersion of GO is also essential for interfacial bonding. Wang et al. [105] found that GO in a particle-adsorbed state could delay the initial hydration reaction of C_3_S but would not hinder the development of hydration products later. GO forms a conformal coating, providing a nucleation barrier for hydrates while inducing the development of denser calcium silicate hydrate fibrils, thus improving the material’s structural performance. To further optimize the dispersion and bonding performance of GO in the cement matrix, Gan et al. [106] introduced nanostabilizers to prepare modified GO (IGO) with high specific surface area and good dispersion. This resulted in a 29.53% increase in compressive strength and a 36.04% increase in the flexural strength of the cement. Additionally, the stability of GO in acidic and highly corrosive environments has received attention. Anwar et al. [28] introduced nanostabilizers to prepare modified GO (IGO) with high specific surface area and good dispersion. This led to a 29.53% improvement in compressive strength and a 36.04% increase in flexural strength of the concrete. Additionally, the stability of GO in acidic and highly corrosive environments has received attention. Anwar et al. studied the durability of concrete exposed to pH 1.0 H_2_SO_4_ baths with different GO dosages and found that high GO dosage significantly enhanced the concrete’s durability under acidic exposure, as indicated by reduced strength loss, lower corrosion depth, and decreased water absorption. These results suggest that GO enhances the mechanical strength of concrete and additionally provides higher durability in harsh environments.

In summary, the role of GO in cementitious materials is closely associated with its dispersion state, the stability of surface functional groups, and its interactions with cement hydration products. Future studies should focus on optimizing GO dispersion and its behavior in complex environments, thus improving the properties of cementitious materials.

## 5. Hybrid Approaches

**Figure 9:**
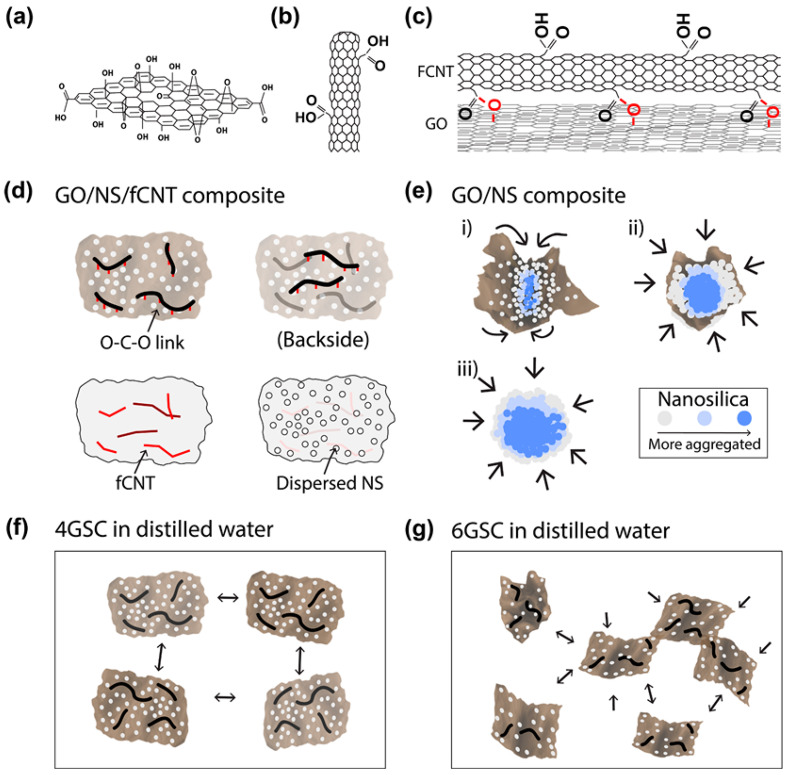
Schematic illustrations of the (**a**) GO, (**b**) FCNTs, (**c**) mechanism of hybrid GO/FCNT reinforcement, (**d**) structure of the triple-hybrid GO/NS/FCNT, (**e**) agglomeration mechanism of GO/NS, and the dispersion morphologies in distilled water of (**f)** 4GSC and (**g**) 6GSC [21].

The integration of 0D or 1D nanomaterials with 2D graphene oxide in cement composites has demonstrated significant synergistic reinforcement effects. Studies have shown that the triple-hybrid structure composed of GO, nano-silica (NS), and functionalized carbon nanotubes (FCNT) combines the excellent wrinkle resistance and dispersibility of GO, the pozzolanic reactivity of NS, and the enhanced mechanical properties of FCNT. Figure 9 illustrates the reinforcement mechanisms of GO and FCNT, the combined GO/FCNT and GO/NS/FCNT structures, the clustering mechanism of GO/NS, and the dispersion morphology of the triple-hybrid GSC. Compared to single reinforcing agents, this hybrid structure has a greater contribution to enhancing the mechanical properties and durability of cementitious materials [21,127]. Suh and colleagues [21] found that incorporating GO/NS/FCNT composites made the pore structure more uniform, decreased the content of large pores exceeding 50 nm, and improved compressive strength both before and after heating when compared to composites containing only GO or GO/NS. Moreover, the triple-hybrid system notably improved the load-bearing and fracture resistance of mortar and concrete samples, showing higher residual strength and durability compared to unmodified cementitious materials [127].

**Figure 10:**
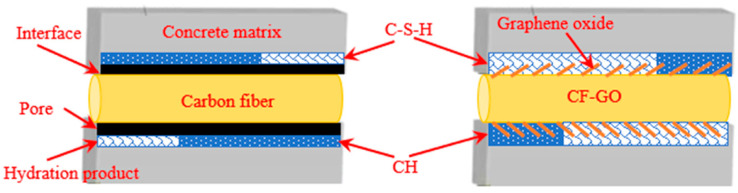
The strengthening effect of CF-GO on the fiber/concrete matrix interface [8].

It has been reported that the strengthening effectiveness of fibers is significantly improved after being coated with graphene oxide. For instance, Wang and colleagues [8] compared the impact of different amounts of graphene oxide grafted carbon fibers (CF-GO) on the performance of cementitious materials, finding that cementitious composites containing 0.3% CF-GO exhibited increases of 24.63%, 27.42%, and 33.21% in compressive, splitting tensile, and bending strength, respectively. This is due to the roughened surface of CF-GO, which increases mechanical interlocking, and the functional groups containing oxygen on the graphene oxide surface acting as nucleation centers for hydration products, attracting their deposition and aggregation [8,64]. The interface enhancement effect is illustrated in Figure 10. Similarly, surface modification of polyvinyl alcohol (PVA) and polyethylene terephthalate (PET) fibers with graphene oxide significantly improved their interface performance with the cement matrix [128,129]. GO formed a tight three-dimensional bond on the surface of PVA fibers, optimizing the interface microstructure and increasing the average indentation modulus and hardness of the reinforced zone by 139% and 435%, respectively. The fracture toughness of hydration products near PVA fibers rose from 0.42 MPa·m^1/2^ to 0.81 MPa·m^1/2^ [128]. For PET fibers, double-coating modification significantly enhanced the mechanical and chemical interactions at the fiber–matrix interface, increasing them to 0.75 MPa and 5.60 J/m^2^, respectively. The tensile strength of PVA-PET fiber-reinforced ECC was also enhanced by this modification, rising from 3.12 MPa to 3.81 MPa, with the tensile strain improving from 1.69% to 2.85% [129]. Furthermore, Zaid et al. [130] found that the combination of 0.09% GO and 2% steel fibers further improved compressive strength, which increased by 39.4% after 90 days of curing compared to the reference samples, demonstrating excellent reinforcement effects and economic feasibility.

To enhance the initial strength of FA-cement composites, researchers incorporated graphene oxide into the FA-cement system to leverage synergistic effects [13,55,57]. Results indicated that graphene oxide optimized the microstructure of FA-GO cement composites by regulating the alignment of hydration crystals, promoting hydration reactions, enhancing the supplementary hydration reactions of FA, refining the pore structure, and reducing porosity, thereby improving their mechanical strength. Yang et al. [57] found that the layered structure of GO and its interaction with FA led to the creation of a denser calcium silicate hydrate structure within the cement matrix, enhancing the load-bearing and bending capacities of cementitious materials.

Graphene oxide, due to its extensive surface area, can act as a medium when combined with different materials, providing multifunctionality to cement composites. Research has shown that even small doses of graphene oxide can improve the electromagnetic shielding efficiency of cement composites. For example, Ullah et al. [84] found that cement composites with 0.03% GO content transmitted 93.57% of the power at 9.3 GHz. Compared to samples without GO, the maximum power transmitted through the sample at 9.3 GHz was approximately 94.15%, effectively enhancing the ability to shield against electromagnetic interference. Additionally, the addition of graphene oxide significantly improved the thermal conductivity of cementitious materials. Zhou and colleagues [14] found that the introduction of GO in phosphogypsum-based self-leveling materials increased thermal conductivity, with the optimal dosage being 1.5 wt%, which resulted in a 17.74% increase in thermal conductivity, enhancing thermal energy storage efficiency. This phenomenon can be attributed to the electronic properties of graphene oxide and its structural role in facilitating heat transfer. The addition of graphene oxide also effectively reduced the resistivity of cement composites, thereby improving their electrical conductivity. For example, the resistivity of concrete decreased by 20% with the addition of 0.025% to 0.1% graphene oxide [131]. Moreover, the combination of graphene oxide with carbon fibers significantly reduced the interconnectivity of the pore system, leading to higher resistivity and further enhancing the electrical performance of the composite [9,13]. In summary, the application of graphene oxide in cement composites not only improves electro-magnetic shielding, thermal conductivity, and electrical conductivity but also enhances their microstructure and durability, showing broad application prospects.

By combining graphene oxide with other functional materials, further research into the versatility and intelligence of cementitious materials can address the demands of advanced engineering infrastructure. This hybrid method improves the mechanical characteristics of the materials while also achieving multifunctional integration, offering new solutions for future engineering applications.

## 6. Conclusions and Prospects

This article provides a comprehensive overview of the latest research progress on the application and reinforcement effects of graphene oxide in cementitious composites. The key findings and future perspectives are as follows:

Graphene oxide tends to aggregate in the high-pH environment of cement pore solutions due to its surface oxygen-containing functional groups, which limits its dispersion and reinforcement efficacy. High-shear mixing can effectively break down GO aggregates into smaller particles. PCE has proven to be an effective dispersant, optimizing GO distribution within the cement matrix through electrostatic repulsion and steric hindrance. The dosage and addition sequence of PCE require further investigation to maximize its dispersing potential. Future research should focus on developing novel surfactants specifically tailored for cement systems to achieve more effective GO dispersion. Additionally, the effects of dispersants on the deoxygenation process of GO warrant deeper exploration.

Incorporating appropriate amounts of GO significantly improves the structural performance, including Young’s modulus and dynamic elastic modulus, of cementitious composites. However, due to the variability in GO properties and the complexity of cementitious systems, research findings remain inconsistent. Current studies predominantly address GO’s effects in cement slurry or mortar, with limited research on its application in concrete, particularly in ultra-fine concrete with low water-to-binder ratios. Expanding research to these areas is crucial for broader practical applications.

GO enhances the durability of cementitious systems by mitigating carbonation, freeze–thaw cycles, and calcium leaching. It achieves this by obstructing the migration of harmful substances through the cement matrix. However, compared to mechanical performance, the mechanisms by which GO improves durability remain underexplored. More in-depth studies are needed to elucidate these mechanisms comprehensively.

The enhancement mechanisms of GO in cementitious materials include its exceptional mechanical properties, promotion of hydration product formation, template effects, and improved interfacial adhesion with the cementitious matrix. These mechanisms synergistically optimize the microstructure of cement composites, leading to enhanced macroscopic performance. However, the specific microstructural changes induced by GO are not yet fully understood, necessitating further detailed investigations.

The integration of GO with other materials can not only enhance the structural performance and durability of cementitious composites but also impart intelligent and multifunctional properties, such as electromagnetic interference shielding, electrical conductivity, and thermal conductivity. Future research should explore innovative combinations of GO with other substances to develop advanced cement composites with superior mechanical properties, extended durability, and multifunctional capabilities for modern construction and infrastructure applications.

In summary, this review consolidates current knowledge on the use of graphene oxide as a nanoscale reinforcement agent in cementitious composites and identifies critical areas for future research. Efforts should prioritize optimizing GO dispersion, investigating its reinforcement mechanisms in various cement systems, and developing multifunctional, intelligent cement composites to meet the demands of advanced engineering and infrastructure projects. Additionally, future research should explore innovative methods for applying ultra-thin GO coatings on aggregates to enhance the mechanical performance of cement-based composites further.

## Figures and Tables

**Table 1:** Preparation methods for cement composites containing GO.

Matrix	w/c ^a^	GO (wt) ^b^	Dispersion Method		Refs.
			Mechanical	Admixture	
Paste	0.5	0.05	Mechanically stirring	-	[46]
Concrete	0.4	0.02	Ultrasonication	FA ^c^, PCE ^d^	[47]
Concrete	0.54	0.02–0.08	Mechanically stirring	PCE	[30]
Mortar	0.35	0.05	-	PCE	[48]
Concrete	0.35	0.08	Mechanically stirring	PCE	[49]
Paste	0.4	0.05	Mechanically stirring	PCE	[26]
Mortar	0.4	0.03	-	PCE	[50]
Mortar	0.5	0.04	Mechanically stirring	PCE	[51]
Paste	0.35	0.002–0.006	Mechanically stirring	PCE, RFP ^e^	[23]
Mortar	0.5	0.02	Mechanically stirring		[11]
Concrete	0.23	0.02–0.06	Mechanically stirring	SP ^f^, steel fiber, SF ^g^	[52]
Paste	0.4	0.05	Mechanically stirring	SP	[37]
Paste	0.6	0.008	Mechanically stirring	FA, SP	[53]
Paste	0.5	0.02–0.08	Mechanically stirring	SP	[38]
Mortar	0.45	0.05	Ultrasonication	SP	[32]
Paste	0.42	0.01–0.05	Mechanically stirring		[54]
Concrete	0.45	0.025, 0.05	Mechanically stirring	FA, SP	[55]
Paste	0.17	0.02–0.06	Mechanically stirring	SP	[56]

^a^: Water-to-cement ratio. ^b^: By weight of cement, the maximum content of GO. ^c^: Fly Ash. ^d^: Polycarboxylate-based superplasticizer. ^e^: Recycled fine powder. ^f^: Superplasticizer. ^g^: Silica fume.

**Table 2:** The influence of GO on the workability of fresh cement composites.

Matrix	w/c	GO Content (wt%) ^a^	Method	The Change of Fluidity/Slump	Refs.
Paste	0.4	0.02	Mini-slump test	The initial and final setting times decreased by 22.2% and 15.9%, respectively.	[72]
Concrete	0.35	0.08	Mini-slump test	The workability decreased by 40%.	[49]
Paste	0.4	0.04	Mini-slump test	The initial setting times decreased by 40.9%.	[73]
Mortar	0.3	0.1	Mini-slump test	The workability decreased by 54%.	[74]
Paste	0.5	0.03	Mini-slump test	The slump flow diameter was 20% lower.	[75]
Paste	0.8	0.03	Slump test	The slump flow was 11.8% lower.	[76]
Paste	0.8	0.15	Mini-slump test	The flowability decreases by 36.7%.	[19]
Concrete	0.45	0.05	Slump cone test	The initial slump reduces by 16.7%.	[55]
Concrete	0.5	0.2	Slump testing	The slump flow was 20% lower.	[70]
Concrete	0.29	0.08	Slump test	The slump flow was 10.2% lower.	[71]
Concrete	0.54	0.08	Slump test	The slump flow was 81% lower.	[30]

^a^: By weight of cement.

**Table 3:** The influence of GO on the mechanical properties of cement-based materials.

Matrix	W/C	Compressive Strength		Flexural Strength		Tensile Strength		Refs.
		GO (wt%) ^a^	Increase (%)/d	GO (wt%)	Increase (%)/d	GO (wt%)	Increase (%)/d	
Mortar	0.5	0.02	24.5/7	0.02	18/7	-	-	[11]
Mortar	0.3	0.1	26.7/28	0.1	32.7/28	0.06	14.2/28	[71]
Paste	0.4	0.02	11.4/28	-	-	-	-	[82]
Paste	0.5			0.06	42.0/28	-	-	[38]
Paste	0.38	0.005	11.9/28	-	-	0.01	17.1/28	[12]
Concrete	0.18	-	-	0.04	14.7/ N.A. ^b^	-	-	[39]
Concrete	0.23	0.04	11.1/ N.A.	-	-	-	-	[52]
Paste	0.4	0.05	9.7/28	0.05	19.4/28	-	-	[26]
Paste	0.35	0.04	17/28	0.04	34.6/28	-	-	[23]
Mortar	0.4	0.06	33.9/28	0.06	47.2/28	-	-	[40]
Concrete	0.54	0.08	21/28	-	-	0.08	12/28	[30]
Concrete	0.54	0.12	33/28	0.12	25/28	0.12	24/28	[18]
Concrete	0.18	0.06	7.7/28	-	-	0.06	28.39/28	[31]
Paste	0.5	0.03	25/28	0.03	20/28	-	-	[72]
Concrete	0.4	0.06	30.0/28	-	-	-	-	[83]
Paste	0.8	0.03	22.1/28	0.03	24.1/28	-	-	[19,73]
Paste	0.45	0.05	57.4/28	0.05	48.2/28	-	-	[84]
Paste	0.4	0.12	12.1/90	0.04	57.3/90	-	-	[63]
Concrete	0.4	0.06	30/28	0.06	33/28	-	-	[76]
Paste	0.4	0.04	9.3/28	-	-	0.04	14.0/28	[85]
Paste	0.45	-	-	-	-	0.025	16/28	[79]
Concrete	0.29	0.06	17.7/28	0.06	21.9/28	-	-	[75]
Paste	0.42	0.03	21.7/28	-	-	-	-	[54]
Concrete	0.45	0.025	10.4/28	0.05	3.6/28	0.05	3.1/28	[55]

^a^: By weight of cement, the optimum dosage of GO. ^b^: Not available.

## Data Availability

No new data were created or analyzed in this study. Data sharing is not applicable to this article.
